# Survivor‐led guidelines for conducting trauma‐informed psychological therapy assessments: Development and modified Delphi study

**DOI:** 10.1111/hex.13585

**Published:** 2022-09-01

**Authors:** Angela Sweeney, Sarah White, Katie Kelly, Alison Faulkner, Stan Papoulias, Steve Gillard

**Affiliations:** ^1^ Service User Research Enterprise, Population Health Research Institute St George's University of London London UK; ^2^ Population Health Research Institute St George's University of London London UK; ^3^ Little Bee Clinic London and University College London London UK; ^4^ Independent Survivor Researcher London UK; ^5^ Service User Research Enterprise, Institute of Psychiatry, Psychology and Neuroscience King's College London London UK; ^6^ School of Health & Psychological Sciences, Population Health Research Institute St George's University of London London UK; ^7^ Present address: Professor of Mental Health, School of Health Sciences University of London London EC1V 0HB UK

**Keywords:** clinical guidelines, Delphi, psychological therapy, service users, trauma‐informed

## Abstract

**Background:**

Psychological therapy assessments are a key point at which a person is accepted into a service or referred on. There is evidence of service users experiencing harm, dropping out of services and potentially experiencing poor outcomes because of inadequate assessment practices. Approaches to assessment tend to be developed by individual services, with a lack of research identifying what makes a good assessment.

**Methods:**

This survivor‐led study, based in England, aimed to generate guidelines for conducting trauma‐informed psychological therapy assessments. The study was guided by a Service User Advisory Group and a Clinician Advisory Group. The study was conducted in three key stages: (i) identifying, modelling and drafting guideline content (ii) modified Delphi study and (iii) guideline finalization. Stage 1 was informed by literature reviews, qualitative research, data workshops with Advisory Groups and an expert consultation. Fifty‐nine people with relevant experiences then participated in a single‐stage modified Delphi (Stage 2). The guidelines were finalized through an analysis of Delphi open comments and a final expert consultation (Stage 3).

**Results:**

The guidelines evolved through each stage of the process, and all items were deemed important by >90% of Delphi participants. The final trauma‐informed guidelines contain eight principles, including ‘focus on relationships’, ‘from systems to people’ and ‘healing environments’.

**Conclusions:**

Experiential knowledge was key in generating the guidelines and conceptualizing content, with a consequent focus on areas, such as recognizing power differentials, understanding oppression as trauma and the relational aspects of assessments. Future research should focus on guideline implementation and investigate whether this impacts service user dropout, engagement with therapy, and outcomes.

**Patient or Public Contribution:**

This study is an example of survivor research, with several authors, including the study lead, identifying as survivors. We consider the ways in which our identities as survivor researchers impacted the study findings.

## 1 INTRODUCTION

In the UK National Health Service (NHS), assessments for psychological therapies—such as cognitive behavioural therapy and psychodynamic psychotherapy—are gateway points at which a decision is taken as to whether a person is accepted into service, declined or referred onwards. Despite the clear importance of the encounter, there are few evidence‐based guidelines for conducting psychological therapy assessments. Instead, assessment processes are typical encounters that are typically developed by individual psychological therapy services, and led by assessors, according to the service modality and setting. Within this, approaches range from the use of highly structured symptom and risk assessment tools^1^ to story‐telling and meaning‐making,^2^ or a combination of both.

There is some evidence that service users can feel harmed by their experiences of psychological therapy assessments.^3^ For instance, structured tools can feel difficult or upsetting^4^, ^5^ and overly technical assessments can result in people's stories becoming reinterpreted, pathologized^6^, ^7^ or viewed through the lens of a particular therapy modality, rather than the person's own sense‐making. Evidence suggests that assessment experiences can be so difficult that they contribute to service drop‐out.^8^ There is also evidence that people who are minoritized, including people from Lesbian, Gay, Bisexual and Transgender and racialized communities, are more likely to experience long‐term harms as a result of therapy,^9^ although there is a lack of research evidence on assessment experiences specifically.^3^ Furthermore, the impact of austerity policies on mental health service provision may compound such harms by prioritizing ‘demand management’^10^, ^11^ and privileging a ‘production‐line mentality’, which undermines the human aspects of healthcare, such as practitioners' ability to convey compassion.^12^ This could occur, for instance, where the focus is on extracting needed information to test eligibility, rather than creating a healing encounter between two people. Nevertheless, clear assessments can support forward therapy engagement, and partially mitigate potential iatrogenic harms—even in cases where the encounter does not result in a therapy place.^13^ In contrast, clear assessments can support forward therapy engagement and partially mitigate the potential iatrogenic harms of therapy.^13^ Findings from one study suggest that positive assessment experiences can be traced to positive therapy outcomes.^14^ Assessments, then, are a key part of the therapeutic process, warranting careful thought and resourcing.

### 1.1 Trauma and psychological therapy assessments

Systematic reviews consistently find an association between childhood trauma—such as bullying, emotional neglect and childhood sexual abuse—and adult mental health outcomes.^15^, ^16^, ^17^ It seems appropriate, therefore, to assume that trauma can play a key role in mental distress and help‐seeking, and that prior experiences of trauma may impact service users' current experiences of psychological therapy assessments.

A small number of studies have explored trauma survivors' experiences of psychological therapies, including some references to assessment processes. Rapsey et al.^4^ interviewed male childhood sexual abuse survivors about their experiences of therapy and found a range of structural barriers to accessing therapy, including the need to undergo assessments. The study also found that failures to ask about childhood sexual abuse, despite this being the primary reason for seeking therapy, contributed to people feeling that their assessors were not listening.

Asking about trauma in assessments can also cause harm. For instance, in research exploring psychological therapy services for people diagnosed with personality disorders, Crawford et al.^18^ found that assessments were experienced as traumatic where trauma was discussed without adequate support being offered afterwards. In their review of the barriers and facilitators to trauma survivors using mental health services, Kantor et al.^19^ found that survivors were often reluctant to enter psychotherapy for fear of re‐experiencing trauma.

Qualitative research undertaken by some of the authors found that experiences of trauma can shape all aspects of psychological therapy assessments, regardless of whether trauma is disclosed.^3^ Through interviews with service users, the study found that while assessments are typically a time of crisis and emotional turbulence for people seeking therapy, assessment encounters can at times be experienced as tick‐box, administrative exercises that leave people feeling like a number to be processed through a system. Where this occurs, an opportunity to create a healing encounter supportive of ongoing service engagement is missed. The research also found that assessments can compound trauma where people are desperate yet feel that the assessor, who is essentially a stranger, has the power to decide whether help is received or not, potentially reinforcing a sense of shame, worthlessness and hopelessness. These damaging experiences, coupled with the dominance of assessor‐led assessment encounters, suggest the need for assessment processes that are able to meet the needs of people with trauma histories.

### 1.2 Trauma‐informed approaches and psychological therapy assessments

Trauma‐informed approaches have arisen in part due to evidence that trauma experiences fundamentally impact survivors' worldviews, relationships and ways of engaging with services and staff.^20^, ^21^ Consequently, in a trauma‐informed service, treatment or therapy is delivered in ways that ensure survivors can engage without experiencing or compounding harms. This approach has been summarized as the ‘four R's’:

A program, organization or system that is trauma‐informed *realizes* the widespread impact of trauma and understands potential paths for recovery; *recognizes* the signs and symptoms of trauma in clients, families, staff and others involved with the system; and *responds* by fully integrating knowledge about trauma into policies, procedures and practices, and seeks to actively *resist re‐traumatization*.^22^ ^,p.9^

In fully integrating knowledge about trauma into service provision, ‘choice, trustworthiness, collaboration and empowerment’ must be facilitated.^23^ Doing so can enable trauma survivors—who may constitute a significant majority of people seeking psychological therapy—to access and remain in services,^20^ preventing drop out and revolving door services. This is particularly important given the often high ‘did not attend’ rates in psychological therapies^24^ and the potential role of assessments within this.^8^

### 1.3 Survivor‐led approaches to research and guideline development

While outcome measures and guidelines have historically been developed by clinicians and academics, recent years have seen growing recognition of the need to include survivor perspectives to centre the needs and priorities of service recipients.^25^ Service user and survivor inclusion in research range from tokenistic consultations through to coproduction and control of the entire research process.^26^ Supportive arguments include the ethical and democratic—such as the need for those who receive services to have a role in shaping them—to the ecological and epistemic—such as the unique validity of knowledge rooted in experience.^27^, ^28^, ^29^

Survivor research—which lies at the control end of the spectrum of involvement—contends that first‐person experiential knowledge is accessed through an exploration of subjective, lived experiences of phenomena.^30^ Reflection on points of connection and disconnection enable a shift from the ‘I’ to the ‘we’,^31^ potentially leading to a form of ‘deep experiential knowledge’.^32^ This can create different ways of understanding phenomena, sometimes challenging taken‐for‐granted knowledge and practices. Reflecting on points of dis/connection and situating these within wider institutional, societal and theoretical levels of understanding^33^ can also mean that survivor research shines a light on the power relations, inequities and intersecting axes of oppression and advantage that shape our lives.^34^, ^35^, ^36^ At the same time, the dominance of positivist epistemologies and the criminalization of knowledge about mental distress (where only clinical contributions to the study and understanding of mental distress are deemed legitimate) tend to relegate survivor‐generated knowledge and survivor researchers to an outsider status within academia.^35^ Survivor researchers have described the multiple barriers that create this ‘outsider status’, including a lack of research departments, centres, chairs and appointments for survivor researchers, and academic institutions, structures, leadership and events that exclude survivors and fail to prioritize the inclusion and leadership of activist scholars.^37^, ^38^ This limits opportunities for, as well as the status (or standing) of, survivor research.

In this survivor‐led research study, we aimed to generate and establish consensus on, guidelines for conducting trauma‐informed psychological therapy assessments, explicitly grounded in knowledge by experience.^27^, ^29^, ^32^

## 2 MATERIALS AND METHODS

### 2.1 Ethics and survivor research

The study was led by the first author, an experienced trauma survivor researcher (A. S.) who has undergone multiple psychological therapy assessments and was guided by two Advisory Groups, a Service User Advisory Group (SUAG) of people with direct experience of undergoing psychological therapy assessments and a Clinician Advisory Group (CAG) whose members had experiences of overseeing, delivering and undergoing psychological therapy assessments. The Advisory Groups were established specifically for this study programme and met approximately twice a year across the 6 years of the study. Advisory Groups contributed to the study design, participated in data workshops and some members chose to contribute as coauthors (K. K. and A. F.). Our approach to engaging with Advisory Groups was influenced by Shimmin et al.'s^39^ account of trauma‐informed, intersectional patient and public involvement, such as utilizing techniques for discursive reflection that create opportunities to consider issues of identity and marginalization.^39^

The research programme was run according to the *Ethics of Survivor Research* outlined by Faulkner.^28^ This meant that as well as prioritizing ethical principles such as clarity transparency and respect, we also worked within a participatory ethos, which aimed to challenge the power asymmetries between researcher and researched, foreground the experiential knowledge of survivors and commit to those service changes most valued by participants. We established a small ethics working group, drawn from the SUAG, to ensure that qualitative research was conducted ethically and safely. This included, for instance, developing a distress protocol, offering an advance copy of interview questions, setting up any questions relating to trauma disclosures carefully before and at interviews and reiterating the right not to respond. Ethics approval was granted by Camberwell and St Giles Research Ethics Committee (18/LO/0077).

### 2.2 Study setting

The study setting was England, United Kingdom, which has publicly funded talking therapy services available via the NHS, with access partially dependent on regional availability. NHS services range from primary care counselling to secondary and tertiary psychotherapy and specialist services in tertiary sites. The United Kingdom also has a national psychological therapy service, Increasing Access to Psychological Therapies (IAPT), which primarily offers interventions based on cognitive behaviour therapy to large numbers of people. Multiple therapy modalities are also available in the private sector.

### 2.3 Stage 1: Identifying, modelling and drafting guideline content

Identifying and drafting the model and content for the guidelines was a multistage process, summarized in Table 1. In Step 1, preliminary work was undertaken to review existing evidence through a systematic and narrative review. Review findings were considered in a data workshop with SUAG members (Step 2) to generate a working model of principles for good practice in trauma‐informed psychological therapy assessments.

**Table 1:** Stage 1: The process to identify, model and draft guideline content

Aim	Steps	Method	Output
Identify content for a model of trauma‐informed psychological therapy assessments	Step 1. Review of existing evidence	a) A systematic review of service users' experiences of psychological therapy assessments. b) A narrative review of trauma‐informed approaches.	Two published reviews.^3^, ^20^
	Step 2. Service User Advisory Group (SUAG) data workshop	A data workshop was held with SUAG members to review potential guideline content extracted from Step 1 and to arrange content into a draft model of trauma‐informed talking therapy assessments.	The SUAG created an early working model of good practice principles for trauma‐informed psychological therapy assessments.
	Step 3. Primary qualitative research	Qualitative interviews with seven service users and seven assessors who had been involved in the same psychological therapy assessment. Participants were recruited from third sector and NHS services (including IAPT), typically with a trauma specialism. Interviews were analysed thematically by the main study researcher (the first author) and members of the SUAG (A. F.) and CAG (K. K.).	Two published qualitative papers.^40^, ^41^
	Step 4. Second SUAG data workshop	SUAG members participated in a second data workshop to discuss key findings and implications for the guidelines.	Discussions were captured and reviewed at Step 5.
Draft guidelines for trauma‐informed talking therapy assessments	Step 5. Assessing the evidence and refining the model	The first author reviewed the work to date (systematic and literature reviews, primary qualitative research and SUAG data workshop) by labelling, relabelling and moving content to create a coherent model that reflected the evidence.	An expanded and refined model of trauma‐informed psychological therapy assessments.
	Step 6. First draft of guidelines	The first author drafted the guidelines based on the refined model of trauma‐informed psychological therapy assessments.	Draft guidelines including each principle with (i) its rationale (ii) key items and (iii) a brief explanation for each item's inclusion.
	Step 7. Expert consultation to review guidelines	Draft guidelines reviewed by seven people with expertize in (i) trauma‐informed approaches, (ii) exploring intersecting experiences of marginalization and (iii) psychological therapy assessments. Members provided extensive written comments on the draft guidelines.	Guidelines were modified by the first author in line with feedback.

Abbreviations: CAG, Clinician Advisory Group; IAPT, Increasing Access to Psychological Therapies; NHS, National Health Service.

Qualitative research was then undertaken with service users and assessors who had been involved in the same psychological therapy assessment (Step 3). Participants were recruited from the third sector and the NHS (including IAPT) psychological therapy services, typically with a trauma specialism, based in a large metropolitan city. Semi‐structured interviews were conducted separately with service users and assessors. Thematic analysis^42^, ^43^ was conducted by the first author, a SUAG member (A. F.) and a CAG member (K. K.*)*, all of whom had lived experience of the topic as well as qualitative expertize; the CAG member had additional experience in conducting assessments. We identified potential guideline items separately and then met to discuss the findings. A second data workshop was then held with the SUAG (Step 4) to discuss key qualitative findings and implications for guideline content.

In Step 5, the first author reviewed findings from Steps 1–4 to familiarize with existing guideline content and further identify items for the developing model of good practice in trauma‐informed psychological therapy. Potential items were extracted from all materials to date, reviewed and grouped into conceptually similar content. Evolving principles were labelled and relabelled, and items reworded and moved, until a coherent model that reflected the evidence base was achieved. Findings were then written into draft guidelines that contained several principles with (i) each principle's rationale, (ii) a series of key items (or statements) and (iii) each item's rationale.

In the final step (Step 6), seven people with expertize in (i) trauma‐informed approaches, (ii) exploring intersecting experiences of marginalization and (iii) psychological therapy assessments participated in an expert consultation. Members received the draft guidelines and provided extensive comments, leading to modifications.

### 2.4 Stage 2: Modified Delphi study

#### 2.4.1 Participants and recruitment

The following stakeholders were approached to participate in the Delphi study:

1. Service users: Including participants in the qualitative study, current users of the services included in the qualitative study, survivor academics and representatives of survivor organizations.
2. Assessors: Including participants in the qualitative study and assessors in the services included in the qualitative study.
3. Other experts: Including academics, policy makers, service managers and commissioners.

Snowballing techniques were used to identify participants, including through Advisory Group member networks, and advertisements in service/organizational newsletters and via specialist counselling services (including for Lesbian, Gay, Bisexual, Transgender and Queer communities, African and Caribbean communities). Participants were predominantly based in the United Kingdom with a small number of international experts also approached.

#### 2.4.2 Data collection

Data collection was conducted using the online survey software LimeSurvey v2.06.^34^ Prospective participants were emailed the survey link, with reminders sent at regular intervals. The survey consisted of two main sections. First, for each guideline principle, a description was given and key items listed. Participants rated the importance of each item on a 0–3 Likert scale, with 0 = *unimportant*, 1 = *not very important*, 2 = *important*, 3 = *very important*. Participants were also able to leave additional comments for each principle and its items. Second, participants gave basic sociodemographic information, including age, gender, ethnicity, country of residence and so on. Participants received a £10 voucher as a thank you for completing the survey.

#### 2.4.3 Data analysis

Frequencies and percentages are used to summarize the sociodemographic data. The findings are summarized by the frequency of items where the median score equalled 3, very important. The minimum value of within‐group consensus (WC) is given for each principle. WC is defined as the % of respondents rating within ±1 point of the median. In calculating WC, when there were missing values, the missing values were assumed to not lie within 1 point of the median.

#### 2.4.4 Delphi stages

Two rounds of data collection and analysis were planned. In Round 1, we anticipated that Delphi scores would inform the ranking, retention or removal of items, with changes confirmed in a second Delphi round.

### 2.5 Stage 3: Guideline finalization

In the final stage, open (qualitative) Delphi comments were imported into Microsoft Word and carefully read by the first author to identify any guideline content that participants considered superfluous or unclear. The content of the guidelines was then changed to address Delphi participants' concerns. The final guidelines were reread by two expert consultation members with specific knowledge of experiences of marginalization and undergoing psychological therapy assessments. Each member read the guidelines and gave detailed comments, with the first author again incorporating the final recommended changes.

## 3 RESULTS

### 3.1 Stage 1: Identifying, modelling and drafting guideline content

The evolution of principles can be seen in Table 2. The findings from the systematic^3^ and narrative^20^ reviews, and the qualitative study^40^ have been published separately. In the first data workshop, the SUAG considered the review findings to create an early working model of trauma‐informed psychological therapy assessments; this contained 7 principles with a total of 32 items (Column 1). This model was then expanded and refined through the identification of further items through the qualitative study and a further data workshop with SUAG members. Following the first author review, a draft model with 7 principles and 129 items (Column 2) was constructed. The expert consultation led to further revision, with the final draft model of trauma‐informed psychological therapy assessments containing 7 principles and 111 items. This model was presented to Delphi participants (Stage 2).

**Table 2:** Development of guideline principles and number of items

Working model of good practice in trauma‐informed psychological therapy assessment generated following literature reviews and Service User Data Workshop (number of items)	Guideline principles reviewed in the first Expert consultation (number of items)	Guideline principles rated by Delphi participants (number of items)	Guideline principles in the final guidelines (number of items)	Example item from final guidelines
There is a ‘need to know’ the information being requested^3^	–	–	Reflections on power^10^	Therapists ensure that they need the information they are asking of people
Therapeutic alliance^5^	Relationships: Trust, clarity and collaboration^20^	Focus on relationships^17^	Focus on relationships^10^	Therapists recognize the expertize that people bring
Clarity of communication and process^4^	From systems to people^21^	From systems to people^13^	From systems to people^10^	There is sufficient time to listen to the person
–	Trauma‐competent, supported assessors^11^	Trauma‐competent, supported therapists^10^	Supported, trauma‐competent, therapists^8^	Therapists receive ongoing training on trauma and trauma‐informed practices
–	Attending to environments^10^	Attending to environments^10^	Healing environments^9^	Sterile and/or clinical environments are avoided or softened
Social identity^3^	Understanding trauma and diversity^9^	Understanding trauma, intersectionalities and oppression^18^	Understanding trauma, intersectionalities and antioppression^12^	Therapists are aware of the potential for an individual's experiences of discrimination and oppression to be pathologized
Safety^6^	Postassessment support^26^	Postassessment support^25^	Postassessment support^16^ and clarity & options when therapy is not offered^4^	Therapists offer to develop a plan with people for the hours and days after the assessment Clear reasons for not being offered therapy are given
Trauma enquiry and disclosure^7^ – Normalizing: acknowledging strengths and coping strategies^4^	Sensitive trauma enquiries and responses^25^	Sensitive trauma enquiries and responses^18^	–	–
	Flexible assessment tools^7^	–	–	–
32 Items	129 Items	111 Items	80 Items	–

### 3.2 Stage 2: Modified Delphi

Fifty‐nine participants responded to the Delphi study, with 52 from the United Kingdom and 3 from the United States (four had a missing country of residence; see Table 3). Fifty percent of participants were aged between 40 and 59 years, and 46 (78%) were female. Forty‐three participants (73%) were White British and five (9%) identified as Black, Asian or Minority Ethnic. Three‐quarters of the sample, 44 (76%), had personal experience of mental distress and/or had used mental health services, and 40 (68%) had an experience of being assessed for talking therapy. Forty‐one (70%) participants work directly with people who experience mental distress and 32 (54%) had an experience conducting talking therapy assessments. Participants worked across multiple sectors: academia, local and national government, charity and other third‐sector organizations and 19 (32%) worked freelance.

**Table 3:** Delphi participants

***n***		**%**
Experience (participants could endorse more than one option)			
Personal experience of mental distress/mental health problems and/or using mental health services		44	75%
Experience of being assessed for a talking therapy		40	68%
Work directly with people who experience mental distress/mental health problems		41	69%
Experience in conducting talking therapy assessments		32	54%
Work in a university or other academic institution		11	19%
Work in a local or national government body		17	29%
Work for a local or national charity or other third‐sector organization		20	34%
Work freelance/independently		19	32%
Country of residence			
UK		56	95%
USA		3	5%
Age (years)			
20–29		4	7%
30–39		11	19%
40–49		15	25%
50–59		15	25%
60–69		11	19%
70+		3	5%
Gender (missing = 1)			
Female		46	78%
Male		12	20%
Ethnicity			
Asian/Asian British Indian		3	5%
Black/Black British Caribbean		1	2%
Mixed heritage White/Asian		1	2%
White British		43	73%
White Irish		2	3%
White Other		9	15%
Sexual orientation			
Bisexual		7	12%
Gay		2	3%
Heterosexual/straight		39	66%
Lesbian		1	2%
Asexual		1	2%
Queer		1	2%
Questioning		1	2%
Prefer not to say		7	12%

The first principle—focus on relationships—had 17 items, 15 (88%) of which had a median score of 3 (indicating very important; see Table 2). The minimum value of WC for this principle was 91.5%. For seven items, the median rating was very important with 100% WC, indicating that all participants rated the items in the principle as important or very important.

From systems to people, the second principle had 13 items of which 4 (31%) had a median rating of 3 (very important). The median score for the remaining items was 2 (important), and the minimum WC value was 94.9%.

The third principle was trauma‐competent, supported assessors. This principle had 10 items; 90% had a median score of 3 and the remaining item had a median of 2. The WC was 88.1% for one item and ranged from 93.2% to 98.3% for the remaining items.

The fourth principle, attending to environments, also contained 10 items. There were slightly more median scores of 2 (60%) than 3 (40%). The WC ranged from 89.8% to 100%. Two items had a median of 3 and a WC value of 100%. Conversely, two items had median scores of 2.

Understanding trauma, intersectionalities and oppression, the fifth principle, was larger than the preceding principles with 18 items. The median score was 3 for 89% of items and 2 for the remaining 2 items. No item was rated as ‘unimportant’. The WC ranged from 89.8% to 100%.

Sensitive enquiries about and responses to trauma, Principle 6, also had 18 items, all of which had median scores of 3. The minimum WC value was 91.5%. Two items had a median score of 3 and a WC value of 100%.

The final principle, postassessment support, contained two parts. The first part of this principle had 20 items, making it the largest. The median score was 3 for 67% of items and 2 for all remaining items. The WC value ranged from 84.7% to 100%. The second part of this principle, if therapy is not offered, contained five items, all with a median score of 3.

### 3.3 Stage 3: Guideline finalization

As the median scores for items did not drop below 2 (important), and the WC values were consistently high, we did not undertake a second, confirmatory Delphi round. Instead, we assessed qualitative comments to select, drop and refine items.

The second expert consultation exercise (with two experts) resulted in three main changes. First, the number of items within each principle was reduced. Second, Principle 6, sensitive enquiries about trauma, was dropped; this was because existing literature gives detailed information on how to make sensitive trauma enquiries, and it was felt that the guidelines should direct people to that literature^44^, ^45^ rather than repeating it. Finally, a new principle was added, ‘moving beyond assessments: reflections on power’, which was further developed through reading key literature.^46^ The final guidelines contain 8 principles with a total of 80 items (see Table 2, columns 4 and 5) (available at: https://www.kcl.ac.uk/ioppn/assets/trauma-informed-assessment-guidelines.pdf).

## 4 DISCUSSION

We engaged in a thorough and extensive survivor researcher‐led process to develop good practice guidelines for trauma‐informed psychological therapy assessments. The final guideline content was deemed important by >90% of Delphi participants, suggesting that the development process was successful in identifying key content. The final guidelines contain eight principles, such as ‘focus on relationships’, ‘from systems to people’, ‘healing environments’ and ‘postassessment support’ (see Table 2, columns 4 and 5 for final principles and example items).

It was notable that the ambition to develop trauma‐informed guidelines was consistently endorsed by study participants: There was felt to be a pressing need for assessment encounters and processes to be shaped by knowledge about trauma and its impacts. Adopting the trauma‐informed guidelines for psychological therapy assessments should enable survivors to engage safely with services, promoting healing from the outset. While incorporating the guidelines should not compromise the quality of services for those without trauma experiences, not incorporating them could mean that trauma survivors are unable to engage with services,^19^ although further research is needed to explore this in more depth.

The survivor‐led process, centring experiential knowledge, led to several shifts over the course of the study in the way that principles were conceptualized. First, rather than understanding assessments as encounters that are inevitably led and directed by assessors, the guidelines shifted towards an understanding of the inherent difficulty for trauma survivors in undergoing assessment and the subsequent need for assessments to be replaced with something more akin to an initial meeting. Our prior qualitative research^40^ found that trauma survivors can feel shame and worthlessness, with the language and processes of assessments compounding these feelings through the knowledge that another person has the power to decide whether help is received or not. Consequently, the final iteration of the guidelines contains the principle, ‘reflections on power’, informed by Proctor's^46^ writing on power in therapy. The principle reflects an understanding of trauma‐informed approaches as ensuring that people have a voice and choice, and are not labelled, judged, diagnosed or assessed.

The second major evolution through the process of generating guideline content was a shift away from the language around social identity and diversity toward the language of oppression. This shift was informed by the expert consultation exercises and key writing on antidiscriminatory practice in therapy by Lago and Smith.^47^ Underpinning this shift was the knowledge that dominant models of UK therapy provision—which are generally white Western in approach—typically fail to understand the role of oppression and discrimination in causing and compounding mental distress and trauma in minoritized groups. Within this, minoritized experiences, such as of racism, homophobia, transphobia and poverty, are considered forms of trauma. Through direct personal experience Sen has observed:

No matter how caring a person is, if they have not experienced or looked into the experience deeply, they won't ‘get’ the trauma, and trauma it is, of having a less than, demonised status.^48^

This highlights the value of adopting a survivor‐led research approach that centralizes experiential knowledge in generating guideline content. Implementing antioppressive practice (which shines a light on the lived experiences and impacts of inequalities and oppressions, and the need for practitioners to understand and respond ethically and respectfully) within a trauma‐informed framework can go some way toward responding to this finding.

Third, development work emphasized the need for trauma‐informed psychological therapy assessments that *focus on relationships* (Principle 2), a common finding in survivor‐led research.^14^ We found that the most healing assessment encounters were those where the assessor communicated their humanity to clients, and this was experienced as authentic, whilst the least healing assessments were those where the assessor was experienced as cold and uncaring. The guidelines reflect this through an understanding of therapeutic relationships as prioritizing trust, honesty, transparency, empathy, collaboration, negotiation, active listening and avoiding pathologization. This can be particularly important for trauma survivors who may have experienced being objectified and treated inhumanly as a core feature of abuse. As Perry and Szalavitz^49^ observe, as trauma is typically experienced relationally, so too is healing.

This is the first study to have generated evidence‐based guidelines for psychological therapy assessments from trauma‐informed and survivor perspectives. Future research should investigate whether giving more time and care to psychological therapy assessments through the implementation of these guidelines creates therapeutic encounters that are more conducive to healing, improve engagement and outcomes, and reduce service dropout.

### 4.1 Strengths and limitations

The survivor‐led process for generating items was robust and extensive, including primary research and literature reviews. This led to >90% consensus in the Delphi. Further key strengths include the relatively large number of Delphi participants and the extensive involvement of a SUAG and a CAG. This use of data workshops with Advisory Groups is novel and increased the validity of findings through multiple voices shaping the analysis and interpretation of data. Engaging in a survivor‐led process focused on experiential knowledge shaped the findings, including the focus on issues of power and oppression.

Limitations include that a modified Delphi approach was employed, which did not include a conventional first round wherein participants generated items (although there was a robust and extensive item development phase); we did not hold a second round due to the high consensus; and as many people hold dual identities, discrete Delphi groups were unachievable. The sampling method may have resulted in a self‐selecting group of participants with a particular perspective on trauma‐informed psychological therapy assessments. Although having a four‐point Likert scale without the option of a mid‐point may have inflated consensus, we attempted to address this by using all qualitative feedback to amend the guidelines. Delphi participants rated 111 items and so scoring may not have been consistent across the exercise due to dipping concentration. However, many participants offered free text comments throughout the consultation, suggesting that engagement remained high. Finally, the study is specific to a UK context; further research into the utility and relevance of the guidelines to other health service systems would be needed before adoption.

## 5 CONCLUSION

Our survivor researcher‐led study generated items for psychological therapy guidelines that were widely endorsed in a modified Delphi. Centring experiential knowledge led to key shifts in the conceptualization of the guidelines' content, including a focus on the recognition of power, understanding trauma through oppression and emphasizing the relational aspects of assessments. Future research should investigate the implementation of the guidelines and any associated impacts on therapy engagement dropout and outcomes.

## AUTHOR CONTRIBUTIONS

*Overall study design*: Angela Sweeney, Steve Gillard. *Qualitative interviews data analysis, Phase 1*: Angela Sweeney, Katie Kelly, Alison Faulkner. *Delphi study design and conduct*: Angela Sweeney and Sarah White. *Writing the first draft of the manuscript*: Angela Sweeney. All authors contributed to, reviewed and agreed to the final version of the manuscript and are accountable for their contributions.

## CONFLICT OF INTEREST

The authors declare no conflict of interest.

## Data Availability

The datasets generated and analysed during the current study are available from the corresponding author on reasonable request.
